# The influence of COVID-19 pandemic on college students’ academic performance and the construction of a learning ability warning system

**DOI:** 10.3389/fpubh.2024.1454406

**Published:** 2024-12-06

**Authors:** Zengqiang Ren, Qiaoling Du

**Affiliations:** ^1^Business School, Northeast Normal University, Changchun, China; ^2^College of Electronic Science and Engineering, Jilin University, Changchun, China

**Keywords:** academic performance, Chinese college student, COVID-19, learning ability warning system, analysis of variance

## Abstract

**Objective:**

This study explored the impact of prevention and control measures taken by Chinese universities on college students’ academic performance during the COVID-19 pandemic.

**Participants:**

The sample includes 1,009 senior students, 1,140 junior and sophomore students, and 1,198 freshman students studying at a top university in China from 2012 to 2023.

**Methods:**

Analysis of variance was used to analyze historical data, and a learning ability warning system based on probabilistic neural networks was further proposed.

**Results:**

There was a significant difference in student academic performance from 2019 to 2022 compared to historical data. Prevention and control measures such as school closures and online teaching have affected the academic performance of college students.

**Conclusion:**

COVID-19 has a negative impact on college students’ learning ability. It is necessary to establish a learning ability warning system to assist university management departments in formulating relevant policies to restore the learning ability of college students.

## 1 Introduction

The outbreak of COVID-19 has had a profound impact on people’s production and life. Economic recession and mobility restrictions have had a negative impact on the physical and mental health of most people (1–4). The epidemic has caused great psychological impact on people, exacerbating psychological problems such as obsessive-compulsive disorder, anxiety, and depression (5). As a special group, college students are usually managed in a centralized manner, with food, accommodation, and transportation all within the scope of the school. During the epidemic, universities have taken a series of prevention and control measures (6), such as school closures (7), closed management of dormitories (8), home quarantine (9) and online teaching (10). These measures have led to adverse psychological stress reactions among different populations, including that of college students, in terms of emotions, academic performance, and other aspects (11–14). The impact of epidemic management measures taken by Chinese universities during the epidemic on student academic performance is a question worthy of in-depth research.

Social cognitive theory holds that learning is a process of continuous interaction between humans and their environment, with personal, behavioral, and environmental factors being the determining factors of human learning (15). A good learning environment can ensure students’ self-discipline, communication and interaction, as well as classroom and after-school learning (16). The lockdown measures taken to prevent the spread of the epidemic have suddenly changed the learning environment of students, greatly altering their psychological state and behavioral patterns. The Austrian government’s ban on people using public places has greatly affected the daily lives and overall wellbeing of Austrians (17). Norwegian universities have almost closed all campus activities and adopted online and digital teaching to prevent the spread of the virus (18). The lockdown measures in the UK have led to a deterioration in the mental health of young people, with a survey showing that 30.3% of people had poor mental health and 10.8% had engaged in self harm (11). The epidemic prevention measures in Africa have led to a serious deterioration of the mental health status of more young people. The report showed that 59.1% of the population was anxiety, 57.5% was worry, 51.5% was depressed, and 22.3% was angry (13). After COVID-19, the level of depression among college students has significantly increased (19). The fatigue caused by prolonged online teaching had a significant impact on academic performance. The level of fatigue was negatively correlated with academic performance, with higher levels of fatigue leading to poorer academic performance (20). Early detection of mental health problems among college students can prevent a decline in their learning ability, and implementing relevant measures can help them recover their learning ability. How to establish a mental health assessment system to identify potential mental health problems early is a worthwhile research topic.

Social cognitive theory holds that learning is a process of continuous interaction between humans and their environment, with personal, behavioral, and environmental factors being the determining factors of human learning (15). A good learning environment can ensure students’ self-discipline, communication and interaction, as well as classroom and after-school learning (16). The lockdown measures taken to prevent the spread of the epidemic have suddenly changed the learning environment of students, greatly altering their psychological state and behavioral patterns. The Austrian government’s ban on people using public places has greatly affected the daily lives and overall wellbeing of Austrians (17). Norwegian universities have almost closed all campus activities and adopted online and digital teaching to prevent the spread of the virus (18). The lockdown measures in the UK have led to a deterioration in the mental health of young people, with a survey showing that 30.3% of people have poor mental health and 10.8% have engaged in self harm (11). The epidemic prevention measures in Africa have led to a serious deterioration of the mental health status of more young people. The report shows that 59.1% of the population presents anxiety, 57.5% presents worry, 51.5% is depressed, and 22.3% is angry (13). After COVID-19, the level of depression among college students has significantly increased (19). The fatigue caused by prolonged online teaching has a significant impact on academic performance. The level of fatigue is negatively correlated with academic performance, indicating that the higher the level of fatigue, the worse the academic performance (20). Early detection of mental health problems among college students can prevent a decline in their learning ability, and implementing relevant measures can help them recover their learning ability. How to establish a mental health assessment system to identify potential mental health problems early is a worthwhile research topic.

Psychological health assessment helps identify potential mental health issues, and evaluation methods include standardized psychological assessment tools (21), statistical analysis (2, 22), model analysis (1, 4, 23–30), and robot learning (31–40). Kettler et al. developed an assessment scale to measure the progress of graduate students in psychology courses in terms of abilities. The content of this scale is based on the Health Service Psychology Accreditation Standards of the American Psychological Association (APA), and uses the Rutgers University Psychology Student Evaluation Tool (RIESP) to analyze the progress of graduate students in terms of abilities (21). However, using statistical analysis of mental health is the simplest and most direct method, and existing evaluation tools cannot meet the needs of mental health analysis in all scenarios. In order to analyze the impact of different factors on mental health, statistical variables were introduced into the evaluation process. Van Der Horst et al. used specified correlation values to reflect the insecurity, anxiety, and pain of spinal surgery patients (22). Mead et al. used a single sample t-test to verify that the measurement of happiness in the current sample based on the Warwick Edinburgh Mental Health Scale significantly decreased (2). The statistical analysis method can provide qualitative analysis basis for mental health from a variable perspective based on the characteristics of statistics.

Model analysis methods can meet the qualitative analysis of mental health from multiple information and perspectives. Rosas Fuentes et al. designed an explanatory model for depression and suicidal ideation based on irrational beliefs of psychological students using multivariate analysis (23). Huang et al. used decision tree models and Kendall correlation analysis to establish a psychological problem prediction model based on objective behavioral data of college students (24). Yao constructed and tested various health indicators of the two factor liquidity model, verifying the applicability of the psychological health two factor liquidity model in the student population (25). Antaramian used a two factor mental health model to analyze the subjective wellbeing and clinical symptom levels of college students, and examined group differences in student engagement and academic performance (26). Tejada Gallardo et al. used network analysis to explore the restructuring of the relationship between school based mental health indicators after MPPI (27). Kim et al. used the Contrastive Avoidance Model (CAM) to verify that concerns increase negative impacts and decrease positive impacts (28). Ye et al. constructed a sustainable happiness model based on emotion action experience outcome using the PERMA model, which showed that positive emotions have a positive impact on engagement and achievement (29). Uzir et al. analyzed the psychological distress of small business owners: depression, anxiety, and stress (DASS-21 scale) using Partial Least Squares Structural Equation Modeling (PLS-SEM). The research results indicate that during the outbreak of COVID-19, DASS-21 parameters and fear of business losses can affect psychological distress (30). Stanculescu et al. examined the psychometric characteristics of the Romanian version of the COVID-19 Fear Scale (FCV-19S) based on classical testing theory and the graded response model, emphasizing the negative correlation between resilience, happiness, and fear in the context of the COVID-19 pandemic (4). Fazia et al. focused on the adverse effects of social isolation on mental health and wellbeing, and used a linear mixed effects model to test the beneficial effects of Integrated Medication (IM) on happiness, perceived stress, and state anxiety (1). Model analysis can address qualitative analysis issues related to specific mental health. However, the multiple parameters of mental health indicators are usually mutual inductance and non-linear, so using model methods cannot further analyze mental health issues in depth.

The prediction of mental health is a non-linear problem, and machine learning can solve complex causal relationships. Wang et al. proposed a multi-layer attention convolutional neural network (ACNN) for text sentiment analysis based on hierarchical CNN. The CNN validated the proposed model’s good positive psychological classification performance in experiments through online physical education teaching resources (31). Yang et al. conducted a detailed analysis and research on the mental health status of college students using data mining clustering methods, and designed an anomaly mining algorithm based on clustering to quickly discover health problems in abnormal data (32). Collins et al. used machine learning to predict individual responses to network-based PPI. Tests have shown that machine learning can be used to predict changes in results of web-based PPI, and it has important clinical significance for matching individuals with PPI based on personal characteristics (33). Sun et al. have developed a long-term and ubiquitous interpretable psychological computing model based on prior knowledge and multimodal information fusion. This model achieves advanced accuracy in basic and complex emotion detection on the proposed mental health database, effectively solving the scientific and accuracy related problems in long-term recognition and prediction of complex mental health states (34). Pan et al. established an emotion classification model to annotate 105,536 epidemic comments on the official Weibo account of People’s Daily within 86 days (positive and negative). The test results showed that the accuracy of the model reached 88%, and the AUC value was greater than 0.9 (3). Li established a DNN based network model for predicting the mental health of enterprise employees. The key influencing factors in mental health assessment are used as input vectors for DNN, and the DNN network model is used to analyze which are the main factors affecting the mental health of enterprise employees. After sample training, the error between the predicted and measured values of DNN was only 3.55%, achieving the expected effect (35). Yao proposed a psychological state analysis model for college students based on BP neural network to analyze the impact of COVID-19 on the mental health of college students. Based on the behavior and psychological characteristics of college students, relevant survey results were obtained through event monitoring, early warning, and daily performance, and a relationship model between the psychological state of college students and the epidemic was constructed based on BP neural network (36). Luo proposed a fuzzy comprehensive evaluation based on BP algorithm and a mental health prediction system for impoverished college students. Using the International Mental Health Scale SCL-90 and considering the mental health status of impoverished college students, an optimized BP algorithm was selected to establish a mental health prediction model, which was implemented and compared with other models to demonstrate its superiority (37). Liu combined psychological knowledge and machine learning methods to propose a psychological crisis warning algorithm based on educational data, which predicts the duration and intensity of emotions by calculating stress events and emotional decay (38). Lin et al. established a CNN based prediction model to predict the tendencies of public emotions and mental health. The proposed method can accurately determine the emotional tendencies of online users (39). Wang et al. constructed a multi task learning model based on pre trained models to detect depression and identify academic performance distortions (40). Machine learning has become a powerful tool for studying mental health. Machine learning can be used to analyze various potential factors, such as school closures, dormitory closures, and online teaching, and their impact on students’ academic performance and mental health. Therefore, this article studies the construction of a college student academic performance warning system based on machine learning methods, which helps schools understand students’ mental health levels from the perspective of their academic performance.

The anxiety caused by the pandemic had a negative impact on students’ academic performance (41). A cross-sectional study was conducted on college students in Anhui Province, China from May to June 2022, and the results showed that academic stress had a significant impact on depression among college students in the context of COVID-19 (*p* < 0.01); College students who have not served as student cadres were more affected by academic pressure (*p* < 0.05); The attitude of college students toward COVID-19 significantly affected depression (*p* < 0.01) (42). A longitudinal study was conducted on Chinese university students from October 2019 to October 2020, and the results showed a significant decline in critical thinking skills and personality from before to after the lockdown. After the lockdown, perceived academic achievements and critical thinking exhibited greater variability. In addition, those who were more susceptible to emotional distress during the pandemic performed poorly in learning outcomes after lockdown (43). The above research indicates that the pandemic has had a negative impact on the academic performance of college students. The cross-sectional and longitudinal studies on Chinese university students support this conclusion, but the longitudinal study only lasted for 2 years, which is insufficient time.

In order to compensate for the short duration of longitudinal research, the data is analyzed in this paper spans from 2012 to 2023. In order to study the impact of the COVID-19 pandemic on the academic performance of college students, this paper first used the ANOVA method to conduct a longitudinal analysis of the final results, to analyze the impact of the epidemic on college students’ academic performance. Secondly, a learning achievement warning system based on probabilistic neural networks was proposed. The data comes from a certain university in China and is the final grades of three professional courses in a certain engineering major, covering the period from 2012 to 2023. The analysis results of this study support the conclusions of (42) and (43). This study provide a reference method for the management of psychological crises among college students, and have a guiding role in the formulation of policies for schools to respond to sudden public health emergencies.

## 2 Academic performance issues among college students in the context of the epidemic

### 2.1 Psychological environment of college students against the background of the epidemic

Through a survey of students’ mental health and personality traits, it was found that the mental health problems of college students mainly focus on five factors: obsessive-compulsive symptoms, emotional imbalance, learning pressure, maladaptation, interpersonal tension and sensitivity. The COVID-19 pandemic has had a great impact on the psychology of college students, exacerbating psychological problems such as obsessive-compulsive disorder, anxiety, depression, interpersonal sensitivity, and also bringing a series of academic performance problems (44).

Firstly, the risk of COVID-19 infection has caused anxiety among college students, with students wearing masks, maintaining social distance, reducing social interactions, and even staying indoors (45). Some college students were unable to study and live normally, resulting in excessive stress reactions. Secondly, during the epidemic, universities had taken strict epidemic prevention measures, but the epidemic continued to spread. College students were passive and weak, feeling powerless and helpless. Finally, the pandemic lasting for 3 years has led to ineffective relief of anxiety, causing college students to experience persistent psychological states such as low mood, pessimism, and helplessness, and their anxiety turns into depression (46, 47). In addition, COVID-19 caused fear among college students, leading them to be afraid to go out, study in the classroom, and interact with others (48). Long term excessive psychological problems such as anxiety, depression, fear, and helplessness among college students not only harmed their physical and mental health, but also affected their academic performance and have a significant negative impact on education and teaching (49, 50).

### 2.2 Learning environment for college students against the background of the epidemic

In order to prevent the COVID-19 epidemic, universities have strengthened centralized management and adopted closed management methods for them, such as closed management of dormitories (8), campus restrictions (7), cafeteria restrictions on dine in, etc. These measures have intensified the fear of the epidemic among college students and further reduced their social interactions.

The sudden closed management has changed the learning and living environment of college students, breaking their study and living habits. The closed management model of the school restricted students’ learning space, resulting in them only being able to study and live in the dormitory. Faced with academic pressure, students were unable to alleviate it through methods such as jogging, swimming, watching movies, and listening to music (51). These measures had led to college students being irritable, impulsive, and emotionally unstable, resulting in adverse psychological stress reactions in cognitive aspects (12, 14, 41).

### 2.3 Teaching methods for college students in the context of the epidemic

In order to stop the spread of COVID-19 to the campus and ensure the safety of teachers and students, the Ministry of Education issued the Guidance of the Leading Group Office of the Ministry of Education in Response to the novel coronavirus Pneumonia Epidemic on Doing a Good Job in the Online Teaching Organization and Management of Ordinary Colleges and Universities during the Period of Epidemic Prevention and Control, calling on all schools around the country to carry out the work of “suspending classes and schools.” It is required to strictly implement the requirements of the technical scheme for the prevention and control of COVID-19 in colleges and universities, primary and secondary schools, and kindergartens issued by the National Health Commission and the Ministry of Education, implement the plan efficiently, and win the first opportunity with speed. According to the requirements of the Ministry of Education, universities have introduced a series of online teaching measures, allowing teachers and students to quickly immerse themselves in unprecedented online teaching models.

Online teaching has changed the learning habits of college students, advocating for self-directed and conscious learning. Under the epidemic prevention and air defense, online teaching in universities included two methods: online teaching at home and online teaching on campus. Home based online teaching adopted a method of home isolation, completing online teaching at home. Compared to traditional classroom teaching, this teaching method lacks a learning atmosphere and requires students to follow the teacher’s teaching process independently, complete knowledge learning in the classroom, and review after class. Online teaching on campus adopts the method of school isolation, and online teaching is completed in the dormitory. Compared to online teaching from home, this teaching method has more stringent restrictions on students. The learning space of students is limited to a smaller space. Classroom knowledge learning and post class review were all completed in the dormitory. Online teaching has brought certain impacts to students, and most students were unable to adapt to the online teaching mode, resulting in certain psychological health problems, including irritability, anxiety, and other emotions (52).

The learning patterns cannot be easily changed, and students’ learning and living habits cannot be easily changed. In response to COVID-19 prevention and control, universities have introduced some policies that have changed the learning and teaching environment of college students, affected their mental health, and had a certain negative impact on their cognition (53).

## 3 The impact of COVID-19 pandemic on academic performance of college students

### 3.1 Data collection

The data comes from a top Chinese university, spanning from 2012 to 2023. These data are the final grades of three major courses for students majoring in engineering at the university. This paper replaces a certain university with the symbol HU, and three engineering courses with the symbols G1, G2, and G3. In the teaching and training plan, G1 is offered for senior students, G2 is offered for junior and sophomore students, and G3 is offered for freshman students. Normally, the number of students for G1, G2, and G3 courses is approximately 100 per year. The statistical data of the students participated in G1, G2, and G3 courses are listed in Table 1, and the statistical data for G1, G2, and G3 courses are listed in Table 2. Note: 1. Due to the adjustment of the teaching syllabus in a certain year, there had been a decrease in the number of elective students for G1 course. 2. In the spring semester of 2013–2014, the teacher of the G2 course went abroad for further studies, so the grades for G2 are missing in Table 2. This is considering that fluctuations in student performance may be influenced by different teachers.

**Table 1:** The characteristics of the students.

Course	Variable		Frequency	Percentage (%)
G1	Sex	Male	791	78.39
		Female	218	21.61
	Age	Below 20 years	16	1.59
		21 years	954	94.54
		Above 22	39	3.87
G2	Sex	Male	896	78.60
		Female	244	21.40
	Age	Below 18 years	18	1.58
		19–20 years	1,077	94.47
		Above 21	45	3.95
G3	Sex	Male	949	79.22
		Female	249	20.78
	Age	Below 17 years	23	1.92
		18 years	1,117	93.24
		Above 19	58	4.84

**Table 2:** Final grade statistics for G1, G2, and G3 from 2012 to 2023.

Academic year	G1					G2					G3				
	*N*	Mean	SD	Min	Max	*N*	Mean	SD	Min	Max	*N*	Mean	SD	Min	Max
2012–2013	102	80.21	9.10	60.00	96.00	101	69.55	13.14	43.00	93.00	102	69.64	19.11	12.00	97.00
2013–2014	101	80.46	7.73	59.00	96.00	–	–	–	–	–	109	73.82	20.40	12.00	99.00
2014–2015	102	78.51	8.45	49.00	98.00	102	58.89	15.23	25.00	91.00	105	69.95	22.79	3.00	99.00
2015–2016	103	81.75	12.23	60.00	98.00	106	65.48	14.14	33.00	94.00	112	72.71	19.31	18.00	98.00
2016–2017	106	81.98	11.12	60.00	99.00	101	68.59	10.47	38.00	96.00	141	74.54	18.38	17.00	100.00
2017–2018	100	83.70	11.98	46.00	99.00	111	61.78	18.17	16.00	94.00	143	82.99	13.05	34.00	100.00
2018–2019	112	79.16	10.82	52.00	100.00	115	60.68	12.72	28.00	84.00	64	80.89	11.20	41.00	97.00
2019–2020	114	78.99	8.66	51.00	94.00	118	51.62	14.58	10.00	86.00	128	80.20	10.84	50.00	98.00
2020–2021	116	65.73	12.58	27.00	98.00	126	48.99	18.09	6.00	91.00	98	83.32	10.16	53.00	99.00
2021–2022	27	87.22	8.46	60.00	95.00	128	50.91	15.91	13.00	88.00	97	77.66	11.26	48.00	98.00
2022–2023	26	90.81	10.82	60.00	100.00	132	64.98	12.74	28.00	89.00	99	75.70	12.02	47.50	98.50
Total	1,009	79.29	11.72	27.00	100.00	1,140	59.83	16.29	6.00	96.00	1,198	76.50	16.70	3.00	100.00

G1, G2, and G3 were courses in the same major’s training plan, and their teaching targets were the same year students, but they were implemented in different academic years. In order to ensure comparability of students in each course (G1, G2, G3) at baseline, this study used randomization methods for analysis of variance and significant differences in academic performance. The specific method is as follows: for each course sample, repeat 100 times using Monte Carlo random sampling, and randomly select 100 samples each time. SPSS software was used to average 100 datasets.

### 3.2 Analysis of variance

One-factor analysis of variance was used to examine the differences in academic performance among college students, according to the year of study of the course. The statistical analysis software is SPSS, and the significance level of One-factor ANOVA is set at 0.05.

**Figure 1:**
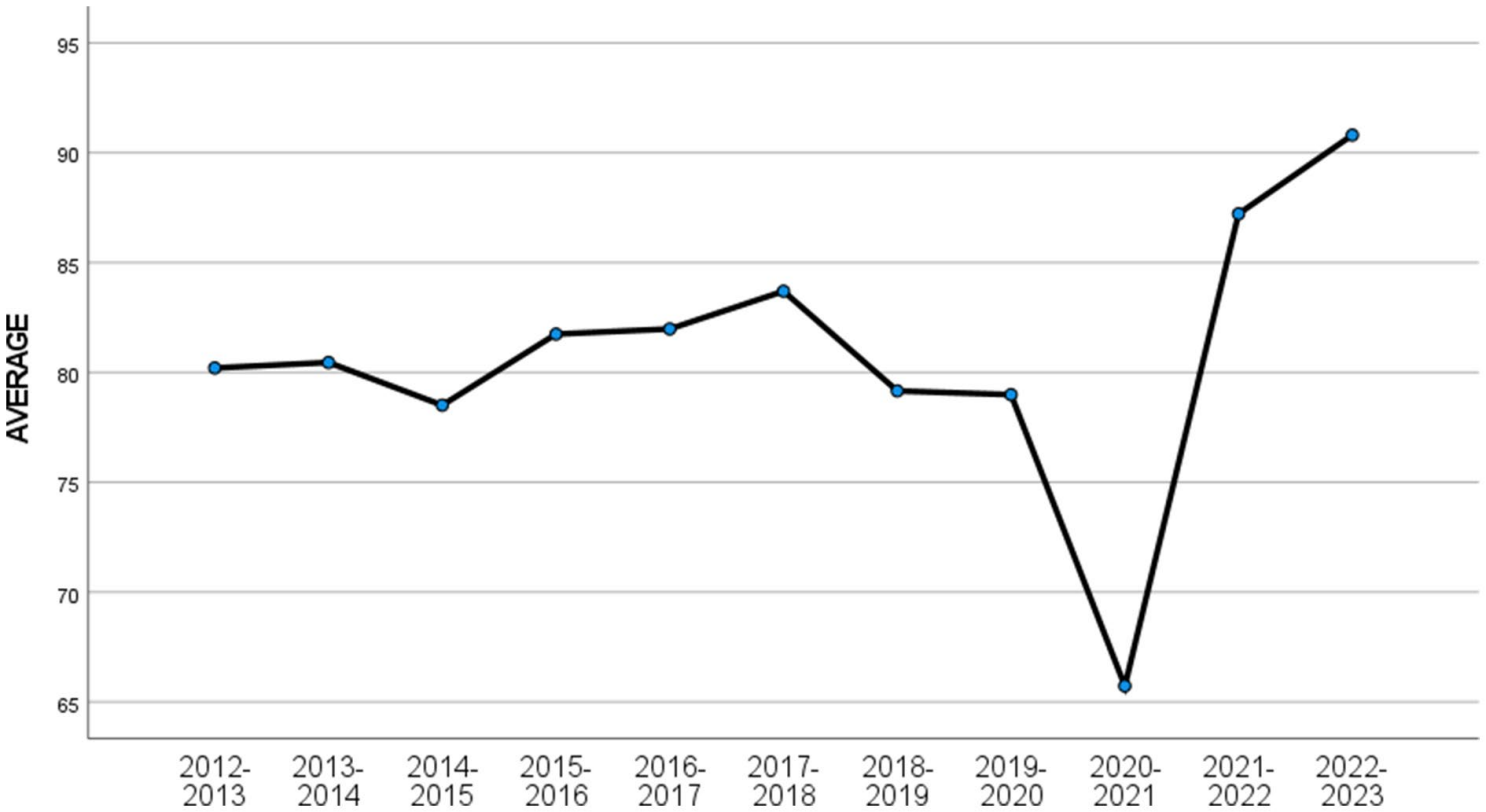
The average annual final grade of G1.

**Figure 2:**
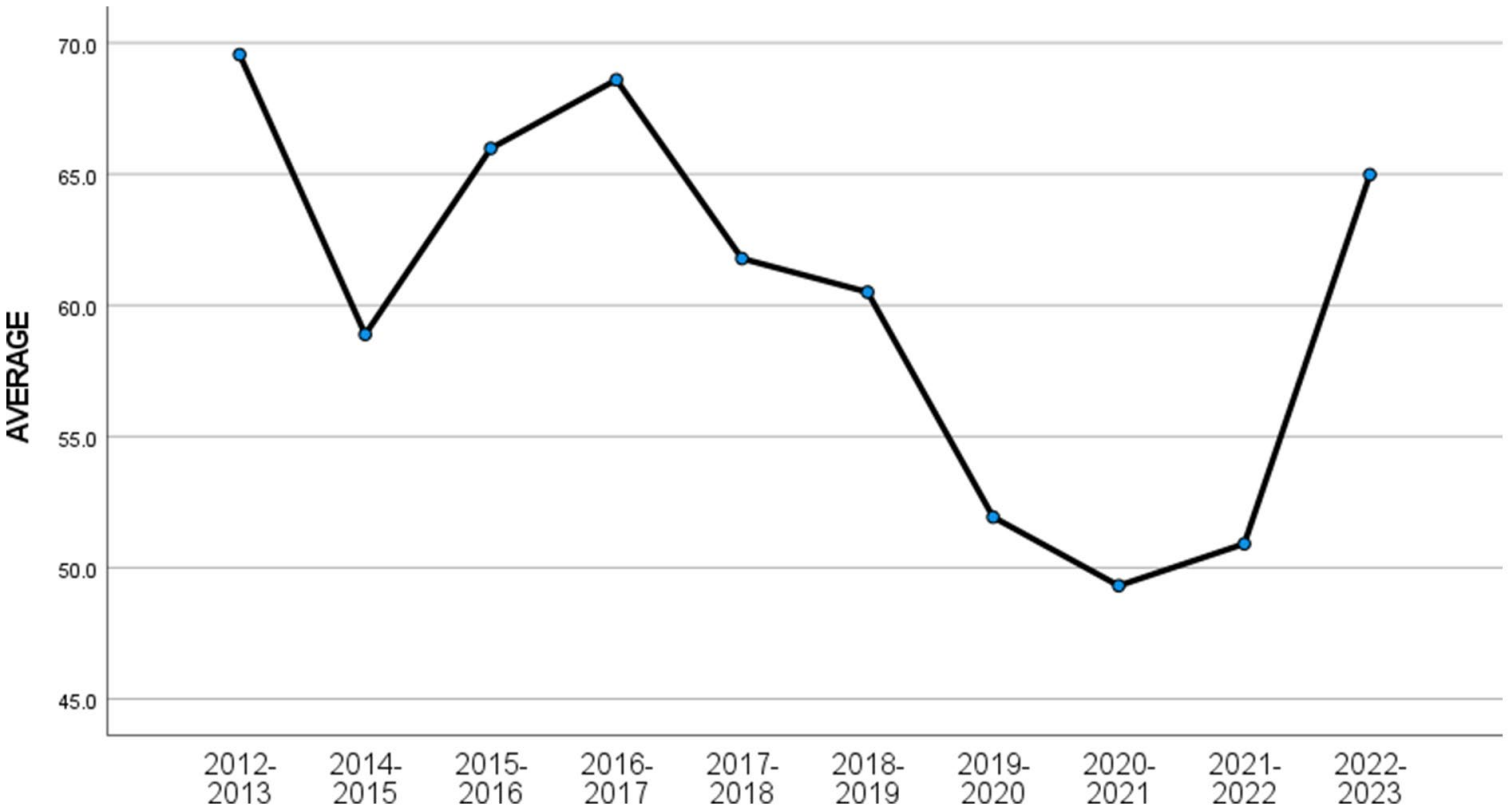
The average annual final grade of G2.

The analysis results of G1, G2, and G3 are shown in Tables 3–6. Table 2 shows the analysis of variance results for G1, G2, and G3. Table 4 shows that there is a significant difference in G1 scores in the 2020–2021 academic year compared to other academic years. Figure 1 shows that the average grade of G1 in the 2020–2021 academic year is the lowest in history. Table 5 shows that there is a significant difference in the final grades of G2 in academic years 2019–2020, 2020–2021, and 2021–2022 compared to the final grades of other academic years. Table 5 shows that the average score of G2 in these three academic years has reached the lowest level in history. Table 6 shows that there is no significant difference in G3 scores for different academic years (Figure 2).

**Table 3:** Analysis of variance of final grades for G1, G2, and G3 over the years.

		Sum of squares	Df	Mean square	*F*	Sig.
G1	Between groups	30,100.03	10	3,010.00	27.70	0.00
	Within groups	108,439.13	998	108.66		
	Total	138,539.17	1,008			
G2	Between groups	56,847.75	9	6,316.42	29.09	0.00
	Within groups	245,346.16	1,130	217.12		
	Total	302,193.91	1,139			
G3	Between groups	25,990.00	10	2,599.00	10.02	0.00
	Within groups	307,886.12	1,187	259.38		
	Total	333,876.12	1,197			

**Table 4:** The significant difference in G1 academic performance between different academic years.

	2012–2013	2013–2014	2014–2015	2015–2016	2016–2017	2017–2018	2018–2019	2019–2020	2020–2021	2021–2022	2022–2023
2012–2013	–										
2013–2014	−0.25	–									
2014–2015	1.70	1.95	–								
2015–2016	−1.54	−1.29	−3.24*	–							
2016–2017	−1.78	−1.53	−3.47*	−0.23	–						
2017–2018	−3.49*	−3.25*	−5.19*	−1.95	−1.72	–					
2018–2019	1.05	1.29	−0.65	2.59	2.82*	4.54*	–				
2019–2020	1.21	1.46	−0.48	2.76	2.99*	4.71*	0.17	–			
2020–2021	14.47*	14.72*	12.78*	16.02*	16.25*	17.97*	13.43*	13.26*	–		
2021–2022	−7.02*	−6.77*	−8.71*	−5.48*	−5.24*	−3.52	−8.06*	−8.23*	−21.49*	–	
2022–2023	−10.60*	−10.35*	−12.30*	−9.06*	−8.83*	−7.11*	−11.65*	−11.82*	−25.08*	−3.59	–

The significance level of the difference in mean values is 0.05.

**Table 5:** The significant difference in G2 academic performance between different academic years.

	2012–2013	2013–2014	2014–2015	2015–2016	2016–2017	2017–2018	2018–2019	2019–2020	2020–2021	2021–2022	2022–2023
2012–2013	–										
2013–2014	–	–	–	–	–	–	–	–	–	–	–
2014–2015	10.66*	–	–								
2015–2016	3.57	–	−7.09*	–							
2016–2017	0.96	–	−9.70*	−2.61	–						
2017–2018	7.77*	–	−2.89	4.20*	6.81*	–					
2018–2019	9.05*	–	−1.61	5.48*	8.09*	1.28	–				
2019–2020	17.62*	–	6.96*	14.05*	16.66*	9.85*	8.57*	–			
2020–2021	20.24*	–	9.57*	16.66*	19.28*	12.47*	11.19*	2.61	–		
2021–2022	18.64*	–	7.98*	15.07*	17.68*	10.87*	9.59*	1.02	−1.60	–	
2022–2023	4.57*	–	−6.09*	1.00	3.61	−3.20	−4.48*	−13.05*	−15.66*	−14.07*	–

The significance level of the difference in mean values is 0.05.

**Table 6:** The significant difference in G3 academic performance between different academic years.

	2012–2013	2013–2014	2014–2015	2015–2016	2016–2017	2017–2018	2018–2019	2019–2020	2020–2021	2021–2022	2022–2023
2012–2013	–										
2013–2014	−4.17	–									
2014–2015	−0.32	3.86	–								
2015–2016	−3.07	1.11	−2.75	–							
2016–2017	−4.90*	−0.72	−4.59*	−1.83	–						
2017–2018	−13.35*	−9.17*	−13.03*	−10.28*	−8.45*	–					
2018–2019	−11.25*	−7.07*	−10.94*	−8.19*	−6.35*	2.10	–				
2019–2020	−10.56*	−6.38*	−10.25*	−7.49*	−5.66*	2.79	0.70	–			
2020–2021	−13.68*	−9.50*	−13.36*	−10.61*	−8.78*	−0.33	−2.43	−3.12	–		
2021–2022	−8.02*	−3.84	−7.71*	−4.95*	−3.12	5.32*	3.23	2.53	5.66*	–	
2022–2023	−6.06*	−1.88	−5.75*	−2.99	−1.16	7.29*	5.19*	4.50*	7.62*	−1.96	–

The significance level of the difference in mean values is 0.05.

## 4 Construction of the learning ability warning system based on probabilistic neural networks

**Figure 3:**
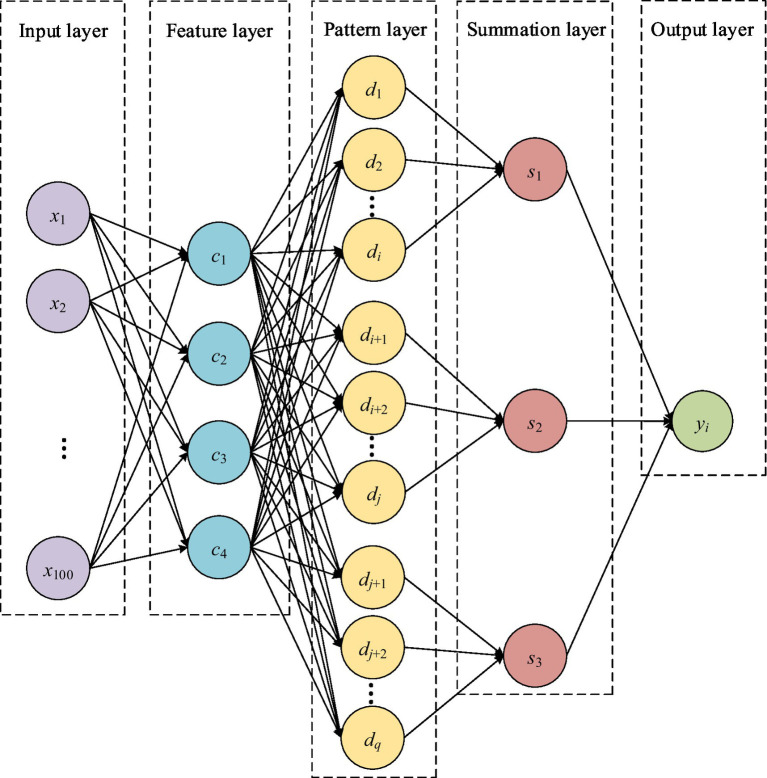
Structure diagram of the learning ability classification system based on probabilistic neural network.

Probabilistic Neural Network (PNN) is a pattern classification neural network model based on statistical principles. This section constructs a learning ability classification system based on probabilistic neural networks to analyze whether there are abnormalities in the learning ability level of college students. This paper divides the learning ability of college students into three levels: normal, degraded, and improved, represented by $Y={\left[1,2,3\right]}^{T}$. The structure of the designed probabilistic neural network is shown in Figure 3, which includes five layers: input layer, feature layer, pattern layer, summation layer, and output layer. The input layer receives the grades, and is set as $X={\left[x_{1},\ldots ,x_{m}\right]}^{T}$. Let m=100, then $x_{i}\;\left(i=1,\cdots ,100\right)$ represents the grades of 100 students. Feature layer extracts feature data from the grades, and is set as $C={\left[c_{1},\ldots ,c_{n}\right]}^{T}$. Let n=4, then $c_{i}\;\left(i=1,\cdots ,4\right)$ represents the maximum, minimum, mean, and standard deviation, respectively. The main function of the pattern layer is to weight and sum the signals output by the feature layer, and send them to the next layer through an activation function. In this paper, the activation function is taken as a Gaussian function. The pattern layer is set as $D={\left[d_{1},d_{2},\ldots ,d_{i},d_{i+1},d_{i+2},\ldots ,d_{j},d_{j+1},d_{j+2},\ldots ,d_{q}\right]}^{T}$, where q represents the number of neurons in the pattern layer, and q is the same as the number of training samples. The sum layer is set as $S={\left[s_{1},s_{2},\ldots ,s_{l}\right]}^{T}$, where l represents the number of neurons in the sum layer, and l is the same as the class value of the training sample. In this system, l=3, then $S={\left[s_{1},s_{2},s_{3}\right]}^{T}$. Finally, the output layer outputs the classification of the sample according to the maximum probability criterion. The definition of $Y={\left[1,2,3\right]}^{T}$ is shown in Table 7.

**Table 7:** College student learning ability level.

*Y*	1	2	3
Level	Improved	Normal	Degraded

**Figure 4:**
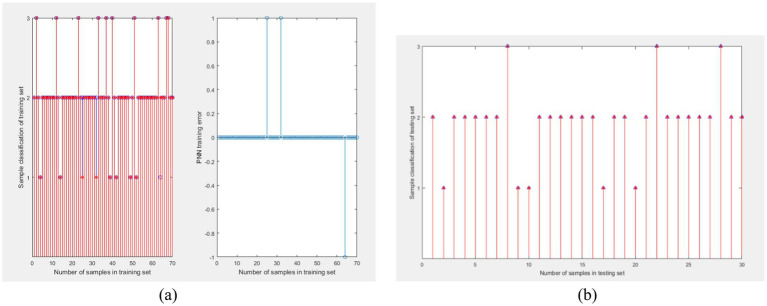
Probability neural network training for G1. **(A)** Training results and **(B)** test results.

The probabilistic neural network for learning ability classification was trained using the final grades of G1, G2, and G3 courses as training data. Due to the different levels of difficulty and teachers in G1, G2, and G3, PNN were trained for each of them, and the training process was the same. During the period of 2012–2023, a total of 1,009 students participated in the course and obtained final grades. These data were used as samples to train probabilistic neural networks. Randomly select 100 out of 1,009 sample data as one sample. Extract 100 times to obtain 100 samples. The ratio of training to testing sets is 7:3. The training results of PNN for G1 are shown in Figure 4A, and the test results are shown in Figure 4B. The PNN was used to classify the ability of college students to learn G1, and the classification results are shown in Table 8.

**Table 8:** Probability neural network prediction results.

G1		G2		G3	
Academic year	Level	Academic year	Level	Academic year	Level
2012–2013	1	2012–2013	1	2012–2013	2
2013–2014	1	2013–2014	–	2013–2014	2
2014–2015	2	2014–2015	2	2014–2015	3
2015–2016	1	2015–2016	1	2015–2016	2
2016–2017	1	2016–2017	1	2016–2017	2
2017–2018	2	2017–2018	2	2017–2018	1
2018–2019	2	2018–2019	1	2018–2019	1
2019–2020	2	2019–2020	3	2019–2020	1
2020–2021	3	2020–2021	3	2020–2021	1
2021–2022	2	2021–2022	3	2021–2022	1
2022–2023	2	2022–2023	1	2022–2023	1

During the period of 2012–2023, a total of 1,140 students participated in the course and obtained final grades. These data were used as samples to train probabilistic neural networks. Randomly select 100 out of 1,140 sample data as one sample. Extract 100 times to obtain 100 samples. The ratio of training to testing sets is 7:3. The training results of PNN for G2 are shown in 0 (a), and the test results are shown in 0 (b). The PNN was used to classify the ability of college students to learn G2, and the classification results are shown in Table 8.

During the period of 2012–2023, a total of 1,198 students participated in the course and obtained final grades. These data were used as samples to train probabilistic neural networks. Randomly select 100 out of 1,198 sample data as one sample. Extract 100 times to obtain 100 samples. The ratio of training to testing sets is 7:3. The training results of PNN for G3 are shown in 0 (a), and the test results are shown in 0 (b). The PNN was used to classify the ability of college students to learn G3, and the classification results are shown in Table 8.

## 5 Discussion

**Figure 5:**
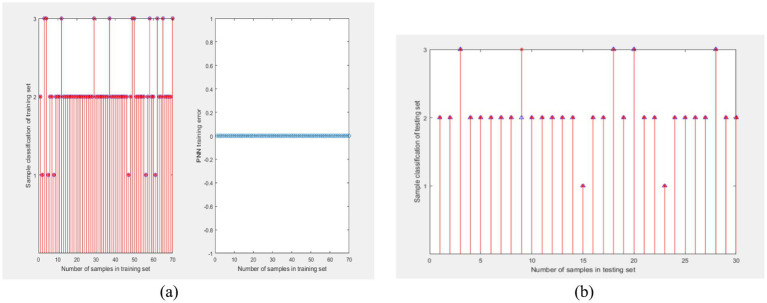
Probability neural network training for G2. **(A)** Training results and **(B)** test results.

**Figure 6:**
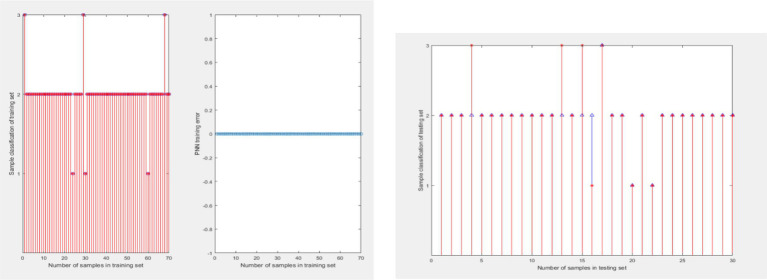
Probability neural network training for G3.

This study investigated the impact of the COVID-19 pandemic on the academic performance of college students. The research sample includes the final grades of three courses for freshmen, sophomores, juniors, and seniors, with a time span of 11 calendar years from 2012 to 2023. The data can reflect the learning ability of students under the background of no epidemic and COVID-19 epidemic (Figures 5, 6).

The researchers noticed that COVID-19 would affect the academic performance of college students (54–57). During the COVID-19 pandemic, the increase in workload for college students, concerns about successfully completing their studies, changes in teaching methods, and the shift toward remote learning have all caused physical and psychological pressure (55). Compared with before the epidemic, academic fatigue, learning engagement, and education satisfaction have all changed, and student happiness and education satisfaction related to learning have decreased by 8% (56). The closure of university campuses and other social isolation measures have had a significant impact on students’ learning environment. Isolation measures have prevented students from participating in regular social activities such as group discussions, quizzes, and brief breaks during lectures, leading to psychological and academic issues (57). In a closed environment, individuals with low psychological resilience may be more emotionally sensitive, and the overall severity of confinement is negatively correlated with depression (58). During the COVID-19 pandemic, most students experienced moderate to high levels of stress related to COVID-19. In addition, some senior students believed that online teaching was a poor choice compared to traditional learning, and their satisfaction with online teaching was relatively low (52). This study provides further evidence for the adverse effects of epidemic prevention measures taken by universities on their academic performance. In our study, the academic performance of second and third grade students was significantly affected by the pandemic. The long-term implementation of epidemic prevention measures (at least 2 years) has greatly affected the mental health level of sophomore, junior, and senior students, leading to a decline in learning ability, which is consistent with (52).

Compared with sophomore, junior, and senior students, the learning ability of freshmen is almost unaffected by the epidemic, which is consistent with (52). This may be due to their new learning environment and unfamiliarity with traditional teaching practices of teachers. The epidemic prevention measures taken by universities, such as campus closures, dormitory closures, and online teaching, have a relatively short implementation time (less than 1 year) for freshmen, resulting in a relatively small impact on their mental health.

In our research, the impact of the epidemic on students’ learning ability comes from objective final grade data. Our research findings confirm that the COVID-19 pandemic has had a negative impact on the learning ability of university students. This conclusion is consistent with the subjective data obtained from the questionnaire survey (59, 60). This is conducive to establishing a learning ability warning system and providing assistance for the formulation of relevant policies. The study also indicates that the designed learning ability warning system based on probabilistic neural networks can predict the learning ability of college students.

This study objectively analyzed the impact of the COVID-19 pandemic on students’ academic performance based on historical data. A student learning ability warning system was constructed using student grades as input variables for the warning system. Compared to the questionnaire survey, the data sample selected in this study has objectivity. Questionnaire survey analysis can reflect the subjective feelings of different groups of people, including college students, toward the COVID-19 pandemic, such as psychological stress, low mood, depression, and anxiety. The questionnaire survey of online teaching can provide feedback information on college students’ satisfaction with online courses, online teaching styles, etc., for research and investigation. However, cross-sectional studies such as questionnaire surveys designed for the COVID-19 pandemic have hindered the inference of causal relationships. The data for this study is from 2012 to 2023, and longitudinal data is beneficial for inferring causal relationships. In addition, objective data analysis is essential for understanding the impact of the COVID-19 pandemic on overall learning success, mental health, and new teaching methods. This study complements this work by objectively analyzing the impact of COVID-19 on college students’ academic performance and building a learning ability early warning system to predict students’ learning situation.

But this study also has limitations. Firstly, our research sample lacks information on the mental and physical condition of participants, which makes it impossible to analyze the causes of certain special situations. Secondly, the sample for this study is only from engineering students, and further research should include students from different majors and universities to analyze the differences between different majors and regions. Finally, this warning system does not include subjective variables such as stress, anxiety, and sleep quality, which should be considered in subsequent research.

The results of this study indicate that sudden changes in teaching methods have affected the learning ability of college students, leading to a decline in their academic performance. The warning system constructed by the research institute provides managers with the current learning status of students based on historical data of their grades, avoiding the problems of long questionnaire survey cycles and poor real-time performance. This provides a feasible method for early detection of changes in students’ learning abilities.

## 6 Conclusion

The epidemic prevention and control measures in universities have effectively controlled the spread of COVID-19 (61). Although college students have well endured the demands of school closures, closed management of dormitories and online teaching, their mental health has been affected to varying degrees. In order to prevent the decline of learning ability among college students, a learning ability warning system should be established to detect problems as early as possible and implement relevant measures to help college students recover their learning ability.

## Data Availability

The raw data supporting the conclusions of this article will be made available by the authors without undue reservation.

## References

[1] Fazia T Bubbico F Nova A Riggi E Caimi G Calgan B. Online short-term mindfulness-based intervention during COVID-19 quarantine in Italy: effects on wellbeing, stress, and anxiety. Front Psychol. (2022) 13:914183. doi: 10.3389/fpsyg.2022.914183, PMID: 35859847 PMC9289612

[2] Mead JP Fisher Z Tree JJ Wong PTP Kemp AH. Protectors of wellbeing during the COVID-19 pandemic: key roles for gratitude and tragic optimism in a UK-based cohort. Front Psychol. (2021) 12:647951. doi: 10.3389/fpsyg.2021.647951, PMID: 34305717 PMC8295471

[3] Pan W Wang RJ Dai WQ Huang G Hu C Pan WL. China public psychology analysis about COVID-19 under considering Sina Weibo data. Front Psychol. (2021) 12:713597. doi: 10.3389/fpsyg.2021.713597, PMID: 34566790 PMC8456024

[4] Stanculescu E. Fear of COVID-19 in Romania: validation of the Romanian version of the fear of COVID-19 scale using graded response model analysis. eCOMMUNITY. (2022) 20:1094–109. doi: 10.1007/s11469-020-00428-4, PMID: 33432266 PMC7787708

[5] Pérez-Escobar JA Carreno DF Pérez-Escobar R Eisenbeck N. Sexual and mental health in healthcare workers during the COVID-19 outbreak: exploring the role of meaning-centered coping. Policy. (2024) 21:1086–99. doi: 10.1007/s13178-024-00963-y

[6] Luo YF Shen HY Yang SC Chen LC. The relationships among anxiety, subjective well-being, media consumption, and safety-seeking behaviors during the COVID-19 epidemic. IJERPH. (2021) 18:13189. doi: 10.3390/ijerph182413189, PMID: 34948796 PMC8700923

[7] Rotvold A Parker K Honrath K Rhee Y. Sleep and diet patterns of college students during the COVID-19 pandemic lockdowns. J Am Coll Heal. (2024) 72:1692–5. doi: 10.1080/07448481.2022.2089850, PMID: 35728122

[8] Sebong PH Tjitradinata C Goldman RE. Promoting COVID-19 prevention strategies in student dormitory setting: a qualitative study. J Am Coll Heal. (2023) 71:1397–406. doi: 10.1080/07448481.2021.1926271, PMID: 34133908

[9] Shanahan ML Fischer IC Rogers SK Rand KL. Coping with COVID-19: a snapshot of college student mental health, coping, and expectancies during stay-at-home orders. J Am Coll Heal. (2024) 72:451–62. doi: 10.1080/07448481.2022.2039670, PMID: 35298356

[10] Hargreaves A Loughnane G Nguyen H Mothersill D. Online learning predictors of mental health in third-level students during the COVID-19 pandemic in Ireland. J Am Coll Heal. (2024) 72:1778–84. doi: 10.1080/07448481.2022.2089852, PMID: 35728258

[11] Dewa LH Crandell C Choong E Jaques J Bottle A Kilkenny C. CCopeY: a mixed-methods coproduced study on the mental health status and coping strategies of young people during COVID-19 UK lockdown. J Adolesc Health Care. (2021) 68:666–75. doi: 10.1016/j.jadohealth.2021.01.009, PMID: 33589305 PMC9188746

[12] Archibong V Usman IM Kasozi KI Aigbogun EO Jr Josiah I Monima AL. Anxiety, anger and depression amongst low-income earners in southwestern Uganda during the COVID-19 Total lockdown. Front Public Health. (2021) 9:590458. doi: 10.3389/fpubh.2021.590458, PMID: 34956994 PMC8695878

[13] Langsi R Osuagwu UL Goson PC Abu EK Mashige KP Ekpenyong B. Prevalence and factors associated with mental and emotional health outcomes among Africans during the COVID-19 lockdown period—a web-based cross-sectional study. IJERPH. (2021) 18:899. doi: 10.3390/ijerph18030899, PMID: 33494209 PMC7908555

[14] Zeng YL Sun WX Wang M Wei JX Sun GC Hu JS. The association between online class-related anxiety and academic achievement among undergraduates. Soc Behav Personal. (2023) 51:e12462:1–13. doi: 10.2224/sbp.12462

[15] Bandura A. Social cognitive theory of mass communication. Media Psychol. (2001) 3:265–99. doi: 10.1207/S1532785xmep0303_03

[16] Zhu JQ Zhao HX Wang X Yang L Qin ZY Geng JC. Effects of online learning on college students in eastern China: a structural equation model. Front Public Health. (2022) 10:853928. doi: 10.3389/fpubh.2022.853928, PMID: 35372181 PMC8968753

[17] Gugerell K Netsch S. Reflection on the Austrian newspaper coverage of the role and relevance of urban open-and green-spaces in Vienna during the first COVID-19 lockdown in 2020. Displays. (2020) 56:54–63. doi: 10.1080/02513625.2020.1906051

[18] Megías-Robles A Gutiérrez-Cobo MJ Cabello R Gómez-Leal R Fernández-Berrocal P. A longitudinal study of the influence of concerns about contagion on negative affect during the COVID-19 lockdown in adults: the moderating effect of gender and resilience. J Health Psychol. (2022) 27:1165–75. doi: 10.1177/1359105321990794, PMID: 33541155 PMC8685745

[19] Morris JIII Morris B Briars A. COVID shelter in place orders and mental health outcomes among college undergraduates. J Am Coll Heal. (2023) 71:2530–7. doi: 10.1080/07448481.2021.1978459, PMID: 34586019

[20] Chen Y Qin XQ. Student fatigue and its impact on teaching effectiveness based on online teaching. Educ Inf Technol. (2024) 29:10177–200. doi: 10.1007/s10639-023-12197-3

[21] Kehler RJ Forman SG Feeney-Kehler KA Haboush KL. Development and initial validation of the Rutgers instrument for evaluation of students in psychology. Train Educ Prof Psychol. (2018) 12:154–62. doi: 10.1037/tep0000200

[22] Van Der Horst A Bohlmeijer ET Schreurs KMG Kelders SM. Strength Back – a qualitative study on the co-creation of a positive psychology digital health intervention for spinal surgery patients. Front Psychol. (2023) 14:1117357. doi: 10.3389/fpsyg.2023.1117357, PMID: 37151334 PMC10160468

[23] Rosas-Fuentes PD Valdés-García KP Monroy-Velasco IR Pérez-Pedraza BD Sánchez-Loyo LM. Depression, suicide ideation, and irrational beliefs: explanatory models in psychology students. Salud Ment. (2023) 46:61–7. doi: 10.17711/Sm.0185-3325.2023.009

[24] Huang YP Li S Lin B Ma S Guo JA Wang CL. Early detection of college students’ psychological problems based on decision tree model. Front Psychol. (2022) 13:946998. doi: 10.3389/fpsyg.2022.946998, PMID: 36033043 PMC9400831

[25] Yao SG. Construction of action model of Students’ mental health education system based on positive psychology. Rev Psicol Deporte. (2020) 29:90–102.

[26] Antaramian S. Assessing psychological symptoms and well-being. J Psychoeduc Assess. (2015) 33:419–29. doi: 10.1177/0734282914557727

[27] Tejada-Gallardo C Blasco-Belled A Alsinet C. Changes in the network structure of mental health after a multicomponent positive psychology intervention in adolescents: a moderated network analysis. Appl Psychol Health Well Being. (2022) 14:987–1003. doi: 10.1111/aphw.12363, PMID: 35466595 PMC9545719

[28] Kim H Newman MG. Worry and rumination enhance a positive emotional contrast based on the framework of the contrast avoidance model. J Anxiety Disord. (2023) 94:102671. doi: 10.1016/j.janxdis.2023.102671, PMID: 36681058 PMC10071830

[29] Ye JH Wu YT Wu YF Chen MY Nong WGJ Lee YS. A study on the construction and validation of pathways to the sustainable well-being of Chinese vocational students in the post-epidemic era. Curr Psychol. (2024) 43:7511–25. doi: 10.1007/s12144-023-04954-x

[30] Uzir MUH Bukari Z Jerin I Hasan N Hamid AA Rannayah T. Impact of COVID‐19 on psychological distress among SME owners in Ghana: partial least square–structural equation modeling (PLS‐SEM) approach. J Community Psychol. (2022) 50:1282–314. doi: 10.1002/jcop.22716, PMID: 34590326

[31] Yang SB Lin L Zhang X. Adjustment method of college students’ mental health based on data analysis under the background of positive psychology. Front Psychol. (2022) 13:921621. doi: 10.3389/fpsyg.2022.921621, PMID: 35846651 PMC9280430

[32] Wang W Hu J. Extraction of PE online teaching resources with positive psychology based on advanced intelligence algorithm. Front Psychol. (2022) 13:948721. doi: 10.3389/fpsyg.2022.948721, PMID: 35899016 PMC9309176

[33] Collins AC Price GD Woodworth RJ Jacobson NC. Predicting individual response to a web-based positive psychology intervention: a machine learning approach. J Posit Psychol. (2024) 19:675–85. doi: 10.1080/17439760.2023.2254743, PMID: 38854972 PMC11156258

[34] Sun X Song YZ Wang M. Toward sensing emotions with deep visual analysis: a long-term psychological modeling approach. IEEE Multimedia. (2020) 27:18–27. doi: 10.1109/Mmul.2020.3025161

[35] Li X. Psychological health assessment model of enterprise employees based on DNN technology. Wirel Commun Mob Comput. (2022) 2022:1–9. doi: 10.1155/2022/4824038

[36] Yao SG. Construction of relationship model between college students’ psychological status and epidemic situation based on BP neural network. Comput Intell Neurosci. (2022) 2022:5115432. doi: 10.1155/2022/5115432, PMID: 35242180 PMC8888073

[37] Luo L. The practice of psychological well-being education model for poor university students from the perspective of positive psychology. Front Psychol. (2022) 13:951668. doi: 10.3389/fpsyg.2022.951668, PMID: 35978785 PMC9376323

[38] Liu LX. The influence of ideological education on students’ mental health during the pandemic: an empirical analysis based on big data and intelligent model. Front Psychol. (2022) 13:940770. doi: 10.3389/fpsyg.2022.940770, PMID: 36312061 PMC9599407

[39] Lin HF Bu NQ. A CNN-based framework for predicting public emotion and multi-level behaviors based on network public opinion. Front Psychol. (2022) 13:909439. doi: 10.3389/fpsyg.2022.909439, PMID: 35814112 PMC9261495

[40] Wang BC Zhao YY Lu X Qin B. Cognitive distortion based explainable depression detection and analysis technologies for the adolescent internet users on social media. Front Public Health. (2023) 10:1045777. doi: 10.3389/fpubh.2022.1045777, PMID: 36733285 PMC9886894

[41] Edo GI Onoharigho FO Nwosu LC Jikah AN Marris DM Akpoghelie PO. Adapting to change: exploring the impact of the COVID-19 pandemic on academic performance in African students and the role of virtual learning as a substitute. J Contin High Educ. (2024):1–17. doi: 10.1080/07377363.2024.2381263

[42] Chen BL Wang WW Yang SL. The relationship between academic stress and depression among college students during the COVID-19 pandemic: a cross-sectional study from China. BMC Psychiatry. (2024) 24:46. doi: 10.1186/s12888-024-05506-8, PMID: 38216950 PMC10785333

[43] Lv XJ Ma JJ Brinthaupt TM Zhao SC Ren XZ. Impacts of university lockdown during the coronavirus pandemic on college students’ academic achievement and critical thinking: a longitudinal study. Front Psychol. (2022) 13:995784. doi: 10.3389/fpsyg.2022.995784, PMID: 36389610 PMC9643715

[44] Garcia C Ayala JA Roldan KD Bavarian N. Exploring Reddit conversations about mental health difficulties among college students during the COVID-19 pandemic. J Am Coll Heal. (2024) 72:2419–25. doi: 10.1080/07448481.2022.2115297, PMID: 36001484 PMC9950288

[45] Wang ZE Bao SS. The impact of social distancing measures (quarantine) policy on tertiary education and medical consultations in China during the COVID-19 pandemic. Front Public Health. (2024) 12:1365805. doi: 10.3389/fpubh.2024.1365805, PMID: 38504676 PMC10948518

[46] Thiria E Pellegrini C Kase BE DeVivo K Steck SE. Health behavior and anxiety changes during the COVID-19 pandemic among students, faculty, and staff at a US university. J Am Coll Heal. (2024) 72:2180–7. doi: 10.1080/07448481.2022.2104615, PMID: 35930456

[47] Vlacha V Perivolaropoulou P. Changes in dietary, lifestyle habits and mood in college students during the COVID-19 pandemic: a survey distributed across Greek universities. J Am Coll Heal. (2024):1–8. doi: 10.1080/07448481.2023.2299428, PMID: 38350002

[48] Baba Z Kienle S Edelblute HB. Using COVID-19 online learning modules to examine concerns of university students returning to in-person learning: a mixed-methods study. J Am Coll Heal. (2024):1–9. doi: 10.1080/07448481.2023.2299407, PMID: 38227923

[49] Shrestha NR Deason RG Cordaro M Howard K Haskard-Zolnierek K. Evaluating the relationship of empathic concern to college students’ responses to the COVID-19 pandemic. J Am Coll Heal. (2023):1–7. doi: 10.1080/07448481.2023.2224432, PMID: 37437177

[50] Quattrone F Borghini A Emdin M Nuti S. Protecting higher education institutions from COVID-19: insights from an Italian experience. J Am Coll Heal. (2022) 70:1354–5. doi: 10.1080/07448481.2020.1791885, PMID: 32701399

[51] Petersen CB Krüger C Guldager JD Algren MH Jervelund SS Berg-Beckhoff G. Are changes in physical activity during COVID-19 associated with mental health among Danish university students? Front Public Health. (2023) 11:1126240. doi: 10.3389/fpubh.2023.1126240, PMID: 37139380 PMC10149910

[52] Tomic SD Tomic S Malenkovic G Malenkovic J Sljivo A Mujicic E. COVID-19-related stress, fear and online teaching satisfaction among nursing students during the COVID-19 pandemic. Healthcare. (2023) 11:894. doi: 10.3390/healthcare11060894, PMID: 36981552 PMC10048461

[53] Jang S Lee H. Changes in Core competencies among Korean university students due to remote learning during the COVID-19 pandemic. Environ Res Public Health. (2021) 18:7476. doi: 10.3390/ijerph18147476, PMID: 34299922 PMC8303327

[54] Fialho PMM Spatafora F Kuhne L Busse H Helmer SM Zeeb H. Perceptions of study conditions and depressive symptoms during the COVID-19 pandemic among university students in Germany: results of the international COVID-19 student well-being study. Front Public Health. (2021) 9:674665. doi: 10.3389/fpubh.2021.674665, PMID: 34178930 PMC8222519

[55] Birmingham WC Wadsworth LL Lassetter JH Graff TC Lauren E Hung M. COVID-19 lockdown: impact on college students’ lives. J Am Coll Heal. (2023) 71:879–93. doi: 10.1080/07448481.2021.1909041, PMID: 34292141

[56] Vollmann M Scheepers RA Nieboer AP Hilverda F. Study-related wellbeing, behavior, and attitudes of university students in the Netherlands during emergency remote teaching in the context of COVID-19: a longitudinal study. Front Psychol. (2022) 13:1056983. doi: 10.3389/fpsyg.2022.1056983, PMID: 36562053 PMC9764013

[57] Strand LB Eilertsen ME Moksnes UK Andre B. Nursing students’ experiences on psychosocial learning environment, personal and social challenges and communication in periods of social isolation: a qualitative study. Inquiry. (2024) 61:00469580241227021. doi: 10.1177/00469580241227021, PMID: 38263715 PMC10807381

[58] de la Rosa PA Cowden RG de Filippis R Jerotic S Nahidi M Ori D. Associations of lockdown stringency and duration with Google searches for mental health terms during the COVID-19 pandemic: a nine-country study. J Psychiatr Res. (2022) 150:237–45. doi: 10.1016/j.jpsychires.2022.03.026., PMID: 35398667 PMC8971703

[59] Baloch GM Sundarasen S Chinna K Nurunnabi M Kamaludin K Khoshaim HB. COVID-19: exploring impacts of the pandemic and lockdown on mental health of Pakistani students. Peerj. (2021) 9:e10612. doi: 10.7717/peerj.10612, PMID: 33604167 PMC7866897

[60] Prowse R Sherratt F Abizaid A Gabrys RL Hellemans KGC Patterson ZR. Coping with the COVID-19 pandemic: examining gender differences in stress and mental health among university students. Psychiatry. (2021) 12:650759. doi: 10.3389/fpsyt.2021.650759, PMID: 33897499 PMC8058407

[61] Allen R Kannangara C Vyas M Carson J. European university students’ mental health during COVID-19: exploring attitudes towards COVID-19 and governmental response. Curr Psychol. (2023) 42:20165–78. doi: 10.1007/s12144-022-02854-0, PMID: 35194357 PMC8831867

