# Supplementary material for: The influence of COVID-19 pandemic on college students’ academic performance and the construction of a learning ability warning system
Source: Front Public Health. 2024 Dec 6;12:1454406. doi: 10.3389/fpubh.2024.1454406 (PMC11659125; doi:10.3389/fpubh.2024.1454406)
Supplement: Supplementary file 1 [file Table_1.docx]

Supplementary Material

# Multiple Comparison of Analysis of Variance for G1

|  | | Mean value difference (I-J) | Standard error | Sig | 95% confidence interval | |
| --- | --- | --- | --- | --- | --- | --- |
|  |  |  |  |  | Lower | 上限 |
| 2012-2013 | 2013-2014 | -0.250 | 1.463 | 0.865 | -3.12 | 2.62 |
|  | 2014-2015 | 1.696 | 1.460 | 0.246 | -1.17 | 4.56 |
|  | 2015-2016 | -1.542 | 1.456 | 0.290 | -4.40 | 1.32 |
|  | 2016-2017 | -1.775 | 1.446 | 0.220 | -4.61 | 1.06 |
|  | 2017-2018 | -3.494^*^ | 1.467 | 0.017 | -6.37 | -0.62 |
|  | 2018-2019 | 1.045 | 1.427 | 0.464 | -1.75 | 3.84 |
|  | 2019-2020 | 1.215 | 1.421 | 0.393 | -1.57 | 4.00 |
|  | 2020-2021 | 14.473^*^ | 1.415 | 0.000 | 11.70 | 17.25 |
|  | 2021-2022 | -7.016^*^ | 2.256 | 0.002 | -11.44 | -2.59 |
|  | 2022-2023 | -10.602^*^ | 2.290 | 0.000 | -15.10 | -6.11 |
| 2013-2014 | 2012-2013 | 0.250 | 1.463 | 0.865 | -2.62 | 3.12 |
|  | 2014-2015 | 1.946 | 1.463 | 0.184 | -0.93 | 4.82 |
|  | 2015-2016 | -1.292 | 1.460 | 0.376 | -4.16 | 1.57 |
|  | 2016-2017 | -1.526 | 1.449 | 0.293 | -4.37 | 1.32 |
|  | 2017-2018 | -3.245^*^ | 1.471 | 0.028 | -6.13 | -0.36 |
|  | 2018-2019 | 1.295 | 1.430 | 0.366 | -1.51 | 4.10 |
|  | 2019-2020 | 1.464 | 1.424 | 0.304 | -1.33 | 4.26 |
|  | 2020-2021 | 14.723^*^ | 1.419 | 0.000 | 11.94 | 17.51 |
|  | 2021-2022 | -6.767^*^ | 2.258 | 0.003 | -11.20 | -2.34 |
|  | 2022-2023 | -10.352^*^ | 2.292 | 0.000 | -14.85 | -5.85 |
| 2014-2015 | 2012-2013 | -1.696 | 1.460 | 0.246 | -4.56 | 1.17 |
|  | 2013-2014 | -1.946 | 1.463 | 0.184 | -4.82 | 0.93 |
|  | 2015-2016 | -3.238^*^ | 1.456 | 0.026 | -6.10 | -0.38 |
|  | 2016-2017 | -3.471^*^ | 1.446 | 0.017 | -6.31 | -0.63 |
|  | 2017-2018 | -5.190^*^ | 1.467 | 0.000 | -8.07 | -2.31 |
|  | 2018-2019 | -0.651 | 1.427 | 0.648 | -3.45 | 2.15 |
|  | 2019-2020 | -0.481 | 1.421 | 0.735 | -3.27 | 2.31 |
|  | 2020-2021 | 12.777^*^ | 1.415 | 0.000 | 10.00 | 15.55 |
|  | 2021-2022 | -8.712^*^ | 2.256 | 0.000 | -13.14 | -4.29 |
|  | 2022-2023 | -12.298^*^ | 2.290 | 0.000 | -16.79 | -7.80 |
| 2015-2016 | 2012-2013 | 1.542 | 1.456 | 0.290 | -1.32 | 4.40 |
|  | 2013-2014 | 1.292 | 1.460 | 0.376 | -1.57 | 4.16 |
|  | 2013-2015 | 3.238^*^ | 1.456 | 0.026 | 0.38 | 6.10 |
|  | 2016-2017 | -0.234 | 1.442 | 0.871 | -3.06 | 2.60 |
|  | 2017-2018 | -1.952 | 1.463 | 0.182 | -4.82 | 0.92 |
|  | 2018-2019 | 2.587 | 1.423 | 0.069 | -0.21 | 5.38 |
|  | 2019-2020 | 2.756 | 1.417 | 0.052 | -0.02 | 5.54 |
|  | 2020-2021 | 16.015^*^ | 1.411 | 0.000 | 13.25 | 18.78 |
|  | 2021-2022 | -5.475^*^ | 2.254 | 0.015 | -9.90 | -1.05 |
|  | 2022-2023 | -9.060^*^ | 2.288 | 0.000 | -13.55 | -4.57 |
| 2016-2017 | 2012-2013 | 1.775 | 1.446 | 0.220 | -1.06 | 4.61 |
|  | 2013-2014 | 1.526 | 1.449 | 0.293 | -1.32 | 4.37 |
|  | 2013-2015 | 3.471^*^ | 1.446 | 0.017 | 0.63 | 6.31 |
|  | 2013-2016 | 0.234 | 1.442 | 0.871 | -2.60 | 3.06 |
|  | 2017-2018 | -1.719 | 1.453 | 0.237 | -4.57 | 1.13 |
|  | 2018-2019 | 2.820^*^ | 1.413 | 0.046 | 0.05 | 5.59 |
|  | 2019-2020 | 2.990^*^ | 1.406 | 0.034 | 0.23 | 5.75 |
|  | 2020-2021 | 16.248^*^ | 1.401 | 0.000 | 13.50 | 19.00 |
|  | 2021-2022 | -5.241^*^ | 2.247 | 0.020 | -9.65 | -0.83 |
|  | 2022-2023 | -8.827^*^ | 2.281 | 0.000 | -13.30 | -4.35 |
| 2017-2018 | 2012-2013 | 3.494^*^ | 1.467 | 0.017 | 0.62 | 6.37 |
|  | 2013-2014 | 3.245^*^ | 1.471 | 0.028 | 0.36 | 6.13 |
|  | 2014-2015 | 5.190^*^ | 1.467 | 0.000 | 2.31 | 8.07 |
|  | 2015-2016 | 1.952 | 1.463 | 0.182 | -0.92 | 4.82 |
|  | 2016-2017 | 1.719 | 1.453 | 0.237 | -1.13 | 4.57 |
|  | 2018-2019 | 4.539^*^ | 1.434 | 0.002 | 1.73 | 7.35 |
|  | 2019-2020 | 4.709^*^ | 1.428 | 0.001 | 1.91 | 7.51 |
|  | 2020-2021 | 17.967^*^ | 1.422 | 0.000 | 15.18 | 20.76 |
|  | 2021-2022 | -3.522 | 2.261 | 0.120 | -7.96 | 0.91 |
|  | 2022-2023 | -7.108^*^ | 2.295 | 0.002 | -11.61 | -2.60 |
| 2018-2019 | 2012-2013 | -1.045 | 1.427 | 0.464 | -3.84 | 1.75 |
|  | 2013-2014 | -1.295 | 1.430 | 0.366 | -4.10 | 1.51 |
|  | 2014-2015 | 0.651 | 1.427 | 0.648 | -2.15 | 3.45 |
|  | 2015-2016 | -2.587 | 1.423 | 0.069 | -5.38 | 0.21 |
|  | 2016-2017 | -2.820^*^ | 1.413 | 0.046 | -5.59 | -0.05 |
|  | 2017-2018 | -4.539^*^ | 1.434 | 0.002 | -7.35 | -1.73 |
|  | 2019-2020 | 0.169 | 1.387 | 0.903 | -2.55 | 2.89 |
|  | 2020-2021 | 13.428^*^ | 1.381 | 0.000 | 10.72 | 16.14 |
|  | 2021-2022 | -8.062^*^ | 2.235 | 0.000 | -12.45 | -3.68 |
|  | 2022-2023 | -11.647^*^ | 2.269 | 0.000 | -16.10 | -7.19 |
| 2019-2020 | 2012-2013 | -1.215 | 1.421 | 0.393 | -4.00 | 1.57 |
|  | 2013-2014 | -1.464 | 1.424 | 0.304 | -4.26 | 1.33 |
|  | 2014-2015 | 0.481 | 1.421 | 0.735 | -2.31 | 3.27 |
|  | 2015-2016 | -2.756 | 1.417 | 0.052 | -5.54 | 0.02 |
|  | 2016-2017 | -2.990^*^ | 1.406 | 0.034 | -5.75 | -0.23 |
|  | 2017-2018 | -4.709^*^ | 1.428 | 0.001 | -7.51 | -1.91 |
|  | 2018-2019 | -0.169 | 1.387 | 0.903 | -2.89 | 2.55 |
|  | 2020-2021 | 13.258^*^ | 1.375 | 0.000 | 10.56 | 15.96 |
|  | 2021-2022 | -8.231^*^ | 2.231 | 0.000 | -12.61 | -3.85 |
|  | 2022-2023 | -11.816^*^ | 2.265 | 0.000 | -16.26 | -7.37 |
| 2020-2021 | 2012-2013 | -14.473^*^ | 1.415 | 0.000 | -17.25 | -11.70 |
|  | 2013-2014 | -14.723^*^ | 1.419 | 0.000 | -17.51 | -11.94 |
|  | 2014-2015 | -12.777^*^ | 1.415 | 0.000 | -15.55 | -10.00 |
|  | 2015-2016 | -16.015^*^ | 1.411 | 0.000 | -18.78 | -13.25 |
|  | 2016-2017 | -16.248^*^ | 1.401 | 0.000 | -19.00 | -13.50 |
|  | 2017-2018 | -17.967^*^ | 1.422 | 0.000 | -20.76 | -15.18 |
|  | 2018-2019 | -13.428^*^ | 1.381 | 0.000 | -16.14 | -10.72 |
|  | 2019-2020 | -13.258^*^ | 1.375 | 0.000 | -15.96 | -10.56 |
|  | 2021-2022 | -21.489^*^ | 2.227 | 0.000 | -25.86 | -17.12 |
|  | 2022-2023 | -25.075^*^ | 2.262 | 0.000 | -29.51 | -20.64 |
| 2021-2022 | 2012-2013 | 7.016^*^ | 2.256 | 0.002 | 2.59 | 11.44 |
|  | 2013-2014 | 6.767^*^ | 2.258 | 0.003 | 2.34 | 11.20 |
|  | 2014-2015 | 8.712^*^ | 2.256 | 0.000 | 4.29 | 13.14 |
|  | 2015-2016 | 5.475^*^ | 2.254 | 0.015 | 1.05 | 9.90 |
|  | 2016-2017 | 5.241^*^ | 2.247 | 0.020 | 0.83 | 9.65 |
|  | 2017-2018 | 3.522 | 2.261 | 0.120 | -0.91 | 7.96 |
|  | 2018-2019 | 8.062^*^ | 2.235 | 0.000 | 3.68 | 12.45 |
|  | 2019-2020 | 8.231^*^ | 2.231 | 0.000 | 3.85 | 12.61 |
|  | 2020-2021 | 21.489^*^ | 2.227 | 0.000 | 17.12 | 25.86 |
|  | 2022-2023 | -3.585 | 2.864 | 0.211 | -9.21 | 2.03 |
| 2022-2023 | 2012-2013 | 10.602^*^ | 2.290 | 0.000 | 6.11 | 15.10 |
|  | 2013-2014 | 10.352^*^ | 2.292 | 0.000 | 5.85 | 14.85 |
|  | 2014-2015 | 12.298^*^ | 2.290 | 0.000 | 7.80 | 16.79 |
|  | 2015-2016 | 9.060^*^ | 2.288 | 0.000 | 4.57 | 13.55 |
|  | 2016-2017 | 8.827^*^ | 2.281 | 0.000 | 4.35 | 13.30 |
|  | 2017-2018 | 7.108^*^ | 2.295 | 0.002 | 2.60 | 11.61 |
|  | 2018-2019 | 11.647^*^ | 2.269 | 0.000 | 7.19 | 16.10 |
|  | 2019-2020 | 11.816^*^ | 2.265 | 0.000 | 7.37 | 16.26 |
|  | 2020-2021 | 25.075^*^ | 2.262 | 0.000 | 20.64 | 29.51 |
|  | 2021-2022 | 3.585 | 2.864 | 0.211 | -2.03 | 9.21 |

The significance level of the difference in mean values is 0.05.

# Multiple Comparison of Analysis of Variance for G2

|  | | Mean value difference (I-J) | Standard error | Sig | 95% confidence interval | |
| --- | --- | --- | --- | --- | --- | --- |
|  |  |  |  |  | Lower | Upper |
| 2012-2013 | 2013-2014 | - | - | - | - | - |
|  | 2014-2015 | 10.6623^*^ | 2.0684 | 0.000 | 6.604 | 14.721 |
|  | 2015-2016 | 3.5733 | 2.0489 | 0.081 | -0.447 | 7.593 |
|  | 2016-2017 | 0.9604 | 2.0735 | 0.643 | -3.108 | 5.029 |
|  | 2017-2018 | 7.7707^*^ | 2.0263 | 0.000 | 3.795 | 11.746 |
|  | 2018-2019 | 9.0501^*^ | 2.0094 | 0.000 | 5.108 | 12.993 |
|  | 2019-2020 | 17.6223^*^ | 1.9974 | 0.000 | 13.703 | 21.541 |
|  | 2020-2021 | 20.2370^*^ | 1.9680 | 0.000 | 16.376 | 24.098 |
|  | 2021-2022 | 18.6404^*^ | 1.9611 | 0.000 | 14.793 | 22.488 |
|  | 2022-2023 | 4.5734^*^ | 1.9480 | 0.019 | 0.751 | 8.395 |
| 2013-2014 | 2012-2013 | - | - | - | - | - |
|  | 2014-2015 | - | - | - | - | - |
|  | 2015-2016 | - | - | - | - | - |
|  | 2016-2017 | - | - | - | - | - |
|  | 2017-2018 | - | - | - | - | - |
|  | 2018-2019 | - | - | - | - | - |
|  | 2019-2020 | - | - | - | - | - |
|  | 2020-2021 | - | - | - | - | - |
|  | 2021-2022 | - | - | - | - | - |
|  | 2022-2023 | - | - | - | - | - |
| 2014-2015 | 2012-2013 | -10.6623^*^ | 2.0684 | 0.000 | -14.721 | -6.604 |
|  | 2013-2014 | - | - | - | - | - |
|  | 2015-2016 | -7.0890^*^ | 2.0438 | 0.001 | -11.099 | -3.079 |
|  | 2016-2017 | -9.7019^*^ | 2.0684 | 0.000 | -13.760 | -5.644 |
|  | 2017-2018 | -2.8916 | 2.0211 | 0.153 | -6.857 | 1.074 |
|  | 2018-2019 | -1.6122 | 2.0042 | 0.421 | -5.544 | 2.320 |
|  | 2019-2020 | 6.9600^*^ | 1.9921 | 0.000 | 3.051 | 10.869 |
|  | 2020-2021 | 9.5747^*^ | 1.9626 | 0.000 | 5.724 | 13.425 |
|  | 2021-2022 | 7.9781^*^ | 1.9557 | 0.000 | 4.141 | 11.815 |
|  | 2022-2023 | -6.0889^*^ | 1.9425 | 0.002 | -9.900 | -2.278 |
| 2015-2016 | 2012-2013 | -3.5733 | 2.0489 | 0.081 | -7.593 | 0.447 |
|  | 2013-2014 | - | - | - | - | - |
|  | 2013-2015 | 7.0890^*^ | 2.0438 | 0.001 | 3.079 | 11.099 |
|  | 2016-2017 | -2.6129 | 2.0489 | 0.202 | -6.633 | 1.407 |
|  | 2017-2018 | 4.1973^*^ | 2.0011 | 0.036 | 0.271 | 8.124 |
|  | 2018-2019 | 5.4768^*^ | 1.9840 | 0.006 | 1.584 | 9.370 |
|  | 2019-2020 | 14.0489^*^ | 1.9719 | 0.000 | 10.180 | 17.918 |
|  | 2020-2021 | 16.6637^*^ | 1.9420 | 0.000 | 12.853 | 20.474 |
|  | 2021-2022 | 15.0671^*^ | 1.9351 | 0.000 | 11.270 | 18.864 |
|  | 2022-2023 | 1.0001 | 1.9218 | 0.603 | -2.771 | 4.771 |
| 2016-2017 | 2012-2013 | -0.9604 | 2.0735 | 0.643 | -5.029 | 3.108 |
|  | 2013-2014 | - | - | - | - | - |
|  | 2013-2015 | 9.7019^*^ | 2.0684 | 0.000 | 5.644 | 13.760 |
|  | 2013-2016 | 2.6129 | 2.0489 | 0.202 | -1.407 | 6.633 |
|  | 2017-2018 | 6.8103^*^ | 2.0263 | 0.001 | 2.835 | 10.786 |
|  | 2018-2019 | 8.0897^*^ | 2.0094 | 0.000 | 4.147 | 12.032 |
|  | 2019-2020 | 16.6619^*^ | 1.9974 | 0.000 | 12.743 | 20.581 |
|  | 2020-2021 | 19.2766^*^ | 1.9680 | 0.000 | 15.415 | 23.138 |
|  | 2021-2022 | 17.6800^*^ | 1.9611 | 0.000 | 13.832 | 21.528 |
|  | 2022-2023 | 3.6130 | 1.9480 | 0.064 | -0.209 | 7.435 |
| 2017-2018 | 2012-2013 | -7.7707^*^ | 2.0263 | 0.000 | -11.746 | -3.795 |
|  | 2013-2014 | - | - | - | - | - |
|  | 2014-2015 | 2.8916 | 2.0211 | 0.153 | -1.074 | 6.857 |
|  | 2015-2016 | -4.1973^*^ | 2.0011 | 0.036 | -8.124 | -0.271 |
|  | 2016-2017 | -6.8103^*^ | 2.0263 | 0.001 | -10.786 | -2.835 |
|  | 2018-2019 | 1.2794 | 1.9606 | 0.514 | -2.567 | 5.126 |
|  | 2019-2020 | 9.8516^*^ | 1.9483 | 0.000 | 6.029 | 13.674 |
|  | 2020-2021 | 12.4663^*^ | 1.9181 | 0.000 | 8.703 | 16.230 |
|  | 2021-2022 | 10.8697^*^ | 1.9111 | 0.000 | 7.120 | 14.619 |
|  | 2022-2023 | -3.1973 | 1.8976 | 0.092 | -6.920 | 0.526 |
| 2018-2019 | 2012-2013 | -9.0501^*^ | 2.0094 | 0.000 | -12.993 | -5.108 |
|  | 2013-2014 | - | - | - | - | - |
|  | 2014-2015 | 1.6122 | 2.0042 | 0.421 | -2.320 | 5.544 |
|  | 2015-2016 | -5.4768^*^ | 1.9840 | 0.006 | -9.370 | -1.584 |
|  | 2016-2017 | -8.0897^*^ | 2.0094 | 0.000 | -12.032 | -4.147 |
|  | 2017-2018 | -1.2794 | 1.9606 | 0.514 | -5.126 | 2.567 |
|  | 2019-2020 | 8.5721^*^ | 1.9308 | 0.000 | 4.784 | 12.361 |
|  | 2020-2021 | 11.1869^*^ | 1.9003 | 0.000 | 7.458 | 14.915 |
|  | 2021-2022 | 9.5903^*^ | 1.8932 | 0.000 | 5.876 | 13.305 |
|  | 2022-2023 | -4.4767^*^ | 1.8796 | 0.017 | -8.165 | -0.789 |
| 2019-2020 | 2012-2013 | -17.6223^*^ | 1.9974 | 0.000 | -21.541 | -13.703 |
|  | 2013-2014 | - | - | - | - | - |
|  | 2014-2015 | -6.9600^*^ | 1.9921 | 0.000 | -10.869 | -3.051 |
|  | 2015-2016 | -14.0489^*^ | 1.9719 | 0.000 | -17.918 | -10.180 |
|  | 2016-2017 | -16.6619^*^ | 1.9974 | 0.000 | -20.581 | -12.743 |
|  | 2017-2018 | -9.8516^*^ | 1.9483 | 0.000 | -13.674 | -6.029 |
|  | 2018-2019 | -8.5721^*^ | 1.9308 | 0.000 | -12.361 | -4.784 |
|  | 2020-2021 | 2.6147 | 1.8876 | 0.166 | -1.089 | 6.318 |
|  | 2021-2022 | 1.0181 | 1.8805 | 0.588 | -2.672 | 4.708 |
|  | 2022-2023 | -13.0489^*^ | 1.8668 | 0.000 | -16.712 | -9.386 |
| 2020-2021 | 2012-2013 | -20.2370^*^ | 1.9680 | 0.000 | -24.098 | -16.376 |
|  | 2013-2014 | - | - | - | - | - |
|  | 2014-2015 | -9.5747^*^ | 1.9626 | 0.000 | -13.425 | -5.724 |
|  | 2015-2016 | -16.6637^*^ | 1.9420 | 0.000 | -20.474 | -12.853 |
|  | 2016-2017 | -19.2766^*^ | 1.9680 | 0.000 | -23.138 | -15.415 |
|  | 2017-2018 | -12.4663^*^ | 1.9181 | 0.000 | -16.230 | -8.703 |
|  | 2018-2019 | -11.1869^*^ | 1.9003 | 0.000 | -14.915 | -7.458 |
|  | 2019-2020 | -2.6147 | 1.8876 | 0.166 | -6.318 | 1.089 |
|  | 2021-2022 | -1.5966 | 1.8492 | 0.388 | -5.225 | 2.032 |
|  | 2022-2023 | -15.6636^*^ | 1.8352 | 0.000 | -19.264 | -12.063 |
| 2021-2022 | 2012-2013 | -18.6404^*^ | 1.9611 | 0.000 | -22.488 | -14.793 |
|  | 2013-2014 | - | - | - | - | - |
|  | 2014-2015 | -7.9781^*^ | 1.9557 | 0.000 | -11.815 | -4.141 |
|  | 2015-2016 | -15.0671^*^ | 1.9351 | 0.000 | -18.864 | -11.270 |
|  | 2016-2017 | -17.6800^*^ | 1.9611 | 0.000 | -21.528 | -13.832 |
|  | 2017-2018 | -10.8697^*^ | 1.9111 | 0.000 | -14.619 | -7.120 |
|  | 2018-2019 | -9.5903^*^ | 1.8932 | 0.000 | -13.305 | -5.876 |
|  | 2019-2020 | -1.0181 | 1.8805 | 0.588 | -4.708 | 2.672 |
|  | 2020-2021 | 1.5966 | 1.8492 | 0.388 | -2.032 | 5.225 |
|  | 2022-2023 | -14.0670^*^ | 1.8279 | 0.000 | -17.653 | -10.481 |
| 2022-2023 | 2012-2013 | -4.5734^*^ | 1.9480 | 0.019 | -8.395 | -0.751 |
|  | 2013-2014 | - | - | - | - | - |
|  | 2014-2015 | 6.0889^*^ | 1.9425 | 0.002 | 2.278 | 9.900 |
|  | 2015-2016 | -1.0001 | 1.9218 | 0.603 | -4.771 | 2.771 |
|  | 2016-2017 | -3.6130 | 1.9480 | 0.064 | -7.435 | 0.209 |
|  | 2017-2018 | 3.1973 | 1.8976 | 0.092 | -0.526 | 6.920 |
|  | 2018-2019 | 4.4767^*^ | 1.8796 | 0.017 | 0.789 | 8.165 |
|  | 2019-2020 | 13.0489^*^ | 1.8668 | 0.000 | 9.386 | 16.712 |
|  | 2020-2021 | 15.6636^*^ | 1.8352 | 0.000 | 12.063 | 19.264 |
|  | 2021-2022 | 14.0670^*^ | 1.8279 | 0.000 | 10.481 | 17.653 |

The significance level of the difference in mean values is 0.05.

# Multiple Comparison of Analysis of Variance for G3

|  | | Mean value difference (I-J) | Standard error | Sig | 95% confidence interval | |
| --- | --- | --- | --- | --- | --- | --- |
|  |  |  |  |  | Lower | 上限 |
| 2012-2013 | 2013-2014 | -4.1793 | 2.2187 | 0.060 | -8.532 | 0.174 |
|  | 2014-2015 | -0.3151 | 2.2390 | 0.888 | -4.708 | 4.078 |
|  | 2015-2016 | -3.0681 | 2.2043 | 0.164 | -7.393 | 1.257 |
|  | 2016-2017 | -4.9018^*^ | 2.0935 | 0.019 | -9.009 | -0.794 |
|  | 2017-2018 | -13.3488^*^ | 2.0873 | 0.000 | -17.444 | -9.254 |
|  | 2018-2019 | -11.2534^*^ | 2.5682 | 0.000 | -16.292 | -6.215 |
|  | 2019-2020 | -10.5581^*^ | 2.1376 | 0.000 | -14.752 | -6.364 |
|  | 2020-2021 | -13.6791^*^ | 2.2781 | 0.000 | -18.149 | -9.210 |
|  | 2021-2022 | -8.0225^*^ | 2.2841 | 0.000 | -12.504 | -3.541 |
|  | 2022-2023 | -6.0617^*^ | 2.2722 | 0.008 | -10.520 | -1.604 |
| 2013-2014 | 2012-2013 | 4.1793 | 2.2187 | 0.060 | -0.174 | 8.532 |
|  | 2014-2015 | 3.8641 | 2.2023 | 0.080 | -0.457 | 8.185 |
|  | 2015-2016 | 1.1112 | 2.1669 | 0.608 | -3.140 | 5.363 |
|  | 2016-2017 | -0.7225 | 2.0541 | 0.725 | -4.753 | 3.308 |
|  | 2017-2018 | -9.1695^*^ | 2.0478 | 0.000 | -13.187 | -5.152 |
|  | 2018-2019 | -7.0741^*^ | 2.5362 | 0.005 | -12.050 | -2.098 |
|  | 2019-2020 | -6.3788^*^ | 2.0991 | 0.002 | -10.497 | -2.261 |
|  | 2020-2021 | -9.4998^*^ | 2.2420 | 0.000 | -13.898 | -5.101 |
|  | 2021-2022 | -3.8433 | 2.2480 | 0.088 | -8.254 | 0.567 |
|  | 2022-2023 | -1.8825 | 2.2360 | 0.400 | -6.269 | 2.504 |
| 2014-2015 | 2012-2013 | 0.3151 | 2.2390 | 0.888 | -4.078 | 4.708 |
|  | 2013-2014 | -3.8641 | 2.2023 | 0.080 | -8.185 | 0.457 |
|  | 2015-2016 | -2.7530 | 2.1877 | 0.209 | -7.045 | 1.539 |
|  | 2016-2017 | -4.5866^*^ | 2.0760 | 0.027 | -8.660 | -0.514 |
|  | 2017-2018 | -13.0336^*^ | 2.0698 | 0.000 | -17.095 | -8.973 |
|  | 2018-2019 | -10.9382^*^ | 2.5540 | 0.000 | -15.949 | -5.927 |
|  | 2019-2020 | -10.2429^*^ | 2.1205 | 0.000 | -14.403 | -6.082 |
|  | 2020-2021 | -13.3639^*^ | 2.2621 | 0.000 | -17.802 | -8.926 |
|  | 2021-2022 | -7.7074^*^ | 2.2681 | 0.001 | -12.157 | -3.257 |
|  | 2022-2023 | -5.7466^*^ | 2.2562 | 0.011 | -10.173 | -1.320 |
| 2015-2016 | 2012-2013 | 3.0681 | 2.2043 | 0.164 | -1.257 | 7.393 |
|  | 2013-2014 | -1.1112 | 2.1669 | 0.608 | -5.363 | 3.140 |
|  | 2013-2015 | 2.7530 | 2.1877 | 0.209 | -1.539 | 7.045 |
|  | 2016-2017 | -1.8336 | 2.0385 | 0.369 | -5.833 | 2.166 |
|  | 2017-2018 | -10.2807^*^ | 2.0322 | 0.000 | -14.268 | -6.294 |
|  | 2018-2019 | -8.1853^*^ | 2.5236 | 0.001 | -13.137 | -3.234 |
|  | 2019-2020 | -7.4900^*^ | 2.0838 | 0.000 | -11.578 | -3.402 |
|  | 2020-2021 | -10.6110^*^ | 2.2277 | 0.000 | -14.982 | -6.240 |
|  | 2021-2022 | -4.9544^*^ | 2.2338 | 0.027 | -9.337 | -0.572 |
|  | 2022-2023 | -2.9936 | 2.2217 | 0.178 | -7.353 | 1.365 |
| 2016-2017 | 2012-2013 | 4.9018^*^ | 2.0935 | 0.019 | 0.794 | 9.009 |
|  | 2013-2014 | 0.7225 | 2.0541 | 0.725 | -3.308 | 4.753 |
|  | 2013-2015 | 4.5866^*^ | 2.0760 | 0.027 | 0.514 | 8.660 |
|  | 2013-2016 | 1.8336 | 2.0385 | 0.369 | -2.166 | 5.833 |
|  | 2017-2018 | -8.4470^*^ | 1.9114 | 0.000 | -12.197 | -4.697 |
|  | 2018-2019 | -6.3516^*^ | 2.4274 | 0.009 | -11.114 | -1.589 |
|  | 2019-2020 | -5.6563^*^ | 1.9662 | 0.004 | -9.514 | -1.799 |
|  | 2020-2021 | -8.7773^*^ | 2.1181 | 0.000 | -12.933 | -4.622 |
|  | 2021-2022 | -3.1208 | 2.1245 | 0.142 | -7.289 | 1.047 |
|  | 2022-2023 | -1.1600 | 2.1118 | 0.583 | -5.303 | 2.983 |
| 2017-2018 | 2012-2013 | 13.3488^*^ | 2.0873 | 0.000 | 9.254 | 17.444 |
|  | 2013-2014 | 9.1695^*^ | 2.0478 | 0.000 | 5.152 | 13.187 |
|  | 2014-2015 | 13.0336^*^ | 2.0698 | 0.000 | 8.973 | 17.095 |
|  | 2015-2016 | 10.2807^*^ | 2.0322 | 0.000 | 6.294 | 14.268 |
|  | 2016-2017 | 8.4470^*^ | 1.9114 | 0.000 | 4.697 | 12.197 |
|  | 2018-2019 | 2.0954 | 2.4221 | 0.387 | -2.657 | 6.848 |
|  | 2019-2020 | 2.7907 | 1.9597 | 0.155 | -1.054 | 6.635 |
|  | 2020-2021 | -0.3303 | 2.1120 | 0.876 | -4.474 | 3.813 |
|  | 2021-2022 | 5.3262^*^ | 2.1185 | 0.012 | 1.170 | 9.483 |
|  | 2022-2023 | 7.2870^*^ | 2.1057 | 0.001 | 3.156 | 11.418 |
| 2018-2019 | 2012-2013 | 11.2534^*^ | 2.5682 | 0.000 | 6.215 | 16.292 |
|  | 2013-2014 | 7.0741^*^ | 2.5362 | 0.005 | 2.098 | 12.050 |
|  | 2014-2015 | 10.9382^*^ | 2.5540 | 0.000 | 5.927 | 15.949 |
|  | 2015-2016 | 8.1853^*^ | 2.5236 | 0.001 | 3.234 | 13.137 |
|  | 2016-2017 | 6.3516^*^ | 2.4274 | 0.009 | 1.589 | 11.114 |
|  | 2017-2018 | -2.0954 | 2.4221 | 0.387 | -6.848 | 2.657 |
|  | 2019-2020 | 0.6953 | 2.4656 | 0.778 | -4.142 | 5.533 |
|  | 2020-2021 | -2.4257 | 2.5884 | 0.349 | -7.504 | 2.653 |
|  | 2021-2022 | 3.2308 | 2.5936 | 0.213 | -1.858 | 8.319 |
|  | 2022-2023 | 5.1916^*^ | 2.5832 | 0.045 | 0.124 | 10.260 |
| 2019-2020 | 2012-2013 | 10.5581^*^ | 2.1376 | 0.000 | 6.364 | 14.752 |
|  | 2013-2014 | 6.3788^*^ | 2.0991 | 0.002 | 2.261 | 10.497 |
|  | 2014-2015 | 10.2429^*^ | 2.1205 | 0.000 | 6.082 | 14.403 |
|  | 2015-2016 | 7.4900^*^ | 2.0838 | 0.000 | 3.402 | 11.578 |
|  | 2016-2017 | 5.6563^*^ | 1.9662 | 0.004 | 1.799 | 9.514 |
|  | 2017-2018 | -2.7907 | 1.9597 | 0.155 | -6.635 | 1.054 |
|  | 2018-2019 | -0.6953 | 2.4656 | 0.778 | -5.533 | 4.142 |
|  | 2020-2021 | -3.1210 | 2.1618 | 0.149 | -7.362 | 1.120 |
|  | 2021-2022 | 2.5355 | 2.1681 | 0.242 | -1.718 | 6.789 |
|  | 2022-2023 | 4.4963^*^ | 2.1556 | 0.037 | 0.267 | 8.725 |
| 2020-2021 | 2012-2013 | 13.6791^*^ | 2.2781 | 0.000 | 9.210 | 18.149 |
|  | 2013-2014 | 9.4998^*^ | 2.2420 | 0.000 | 5.101 | 13.898 |
|  | 2014-2015 | 13.3639^*^ | 2.2621 | 0.000 | 8.926 | 17.802 |
|  | 2015-2016 | 10.6110^*^ | 2.2277 | 0.000 | 6.240 | 14.982 |
|  | 2016-2017 | 8.7773^*^ | 2.1181 | 0.000 | 4.622 | 12.933 |
|  | 2017-2018 | 0.3303 | 2.1120 | 0.876 | -3.813 | 4.474 |
|  | 2018-2019 | 2.4257 | 2.5884 | 0.349 | -2.653 | 7.504 |
|  | 2019-2020 | 3.1210 | 2.1618 | 0.149 | -1.120 | 7.362 |
|  | 2021-2022 | 5.6565^*^ | 2.3067 | 0.014 | 1.131 | 10.182 |
|  | 2022-2023 | 7.6173^*^ | 2.2949 | 0.001 | 3.115 | 12.120 |
| 2021-2022 | 2012-2013 | 8.0225^*^ | 2.2841 | 0.000 | 3.541 | 12.504 |
|  | 2013-2014 | 3.8433 | 2.2480 | 0.088 | -0.567 | 8.254 |
|  | 2014-2015 | 7.7074^*^ | 2.2681 | 0.001 | 3.257 | 12.157 |
|  | 2015-2016 | 4.9544^*^ | 2.2338 | 0.027 | 0.572 | 9.337 |
|  | 2016-2017 | 3.1208 | 2.1245 | 0.142 | -1.047 | 7.289 |
|  | 2017-2018 | -5.3262^*^ | 2.1185 | 0.012 | -9.483 | -1.170 |
|  | 2018-2019 | -3.2308 | 2.5936 | 0.213 | -8.319 | 1.858 |
|  | 2019-2020 | -2.5355 | 2.1681 | 0.242 | -6.789 | 1.718 |
|  | 2020-2021 | -5.6565^*^ | 2.3067 | 0.014 | -10.182 | -1.131 |
|  | 2022-2023 | 1.9608 | 2.3009 | 0.394 | -2.553 | 6.475 |
| 2022-2023 | 2012-2013 | 6.0617^*^ | 2.2722 | 0.008 | 1.604 | 10.520 |
|  | 2013-2014 | 1.8825 | 2.2360 | 0.400 | -2.504 | 6.269 |
|  | 2014-2015 | 5.7466^*^ | 2.2562 | 0.011 | 1.320 | 10.173 |
|  | 2015-2016 | 2.9936 | 2.2217 | 0.178 | -1.365 | 7.353 |
|  | 2016-2017 | 1.1600 | 2.1118 | 0.583 | -2.983 | 5.303 |
|  | 2017-2018 | -7.2870^*^ | 2.1057 | 0.001 | -11.418 | -3.156 |
|  | 2018-2019 | -5.1916^*^ | 2.5832 | 0.045 | -10.260 | -0.124 |
|  | 2019-2020 | -4.4963^*^ | 2.1556 | 0.037 | -8.725 | -0.267 |
|  | 2020-2021 | -7.6173^*^ | 2.2949 | 0.001 | -12.120 | -3.115 |
|  | 2021-2022 | -1.9608 | 2.3009 | 0.394 | -6.475 | 2.553 |

The significance level of the difference in mean values is 0.05.
